# Subcoronary Implantation of Decellularized Aortic Allografts—An Ex Vivo and In Vivo Feasibility Study

**DOI:** 10.1016/j.atssr.2026.01.006

**Published:** 2026-01-22

**Authors:** Murat Avsar, Tomislav Cvitkovic, Alexander Weymann, Klaus Hoeffler, Dmitry Bobylev, Dietmar Boethig, Arjang Ruhparwar, Samir Sarikouch, Alexander Horke

**Affiliations:** Department for Cardiothoracic, Transplant, and Vascular Surgery, Hannover Medical School, Hanover, Germany

## Abstract

**Background:**

Decellularized aortic homografts were introduced in 2008 as a further option for pediatric aortic valve replacement. Subcoronary decellularized aortic homograft implantation may eliminate the need for aortic root replacement and coronary artery reimplantation in patients with sufficient left ventricular outflow tract dimensions.

**Methods:**

Ovine aortic xenografts were harvested from the slaughterhouse and decellularized using an approved detergent-based decellularization protocol. Two cardiac surgeons performed subcoronary implantation in explanted ovine hearts, and results were compared regarding procedure time and procedural success. After these ex vivo experiments, 4 adult black-headed sheep were operated on in acute experiments.

**Results:**

The 2 surgeons were able to perform the ex vivo subcoronary implantation of the decellularized ovine aortic allografts (DOAA) without technical problems within 1 hour. Mechanical strength of the processed ovine DOAA appeared comparable to that of native aortic tissue. Both surgeons stated that there were no differences with respect to appearance, haptics, trimability, and sewability. The initial 2 sheep operated on showed significant valvular incompetence. After modification of the surgical protocol to a single-line suture technique, the next 2 sheep showed fully competent aortic valves with laminar flow across the subcoronary implanted DOAA.

**Conclusions:**

We have developed a technique for subcoronary implantation of decellularized aortic homografts including acute large animal experiments. Long-term animal experiments will provide further insight into durability and recellularization.

**In Short**

▪ Subcoronary implantation of decellularized homograft valves could avoid the need for lifelong anticoagulation and coronary artery transfer.
▪ A new subcoronary implantation technique using decellularized homografts has been developed and tested in acute large-animal experiments.
▪ Using a trimmed aortic valve for subcoronary implantation may further reduce antigen load, potentially minimizing immune response.

Subcoronary implantation of a human aortic valve for aortic valve replacement was pioneered by Donald N. Ross and Brian G. Barratt-Boyes and almost simultaneously published in 1962.^1^^,^^2^ Since then, there have been numerous reports on the subcoronary Ross technique, which is technically demanding but shows excellent long-term results.^3^

With improvements in the manufacturing of mechanical and xenogenic biological valve prostheses, aortic homografts are nowadays used only in selected patients. The main indication presently is reconstruction of the aortic root in extended aortic valve endocarditis or repair of congenital malformations of the left ventricular outflow tract and the aortic valve. Decellularized homografts are a relatively new option for aortic valve and root replacement used in pediatric patients and young adults, showing good results with a freedom from explantation of 67% at 10 years in pediatric patients.^4^

Calcification of the homograft wall appears less pronounced than in conventional cryopreserved allografts; nevertheless, these calcifications may indicate a permanent low-level immune response to decellularized homografts. Current hypotheses point to altered proteins of the extracellular matrix, so-called matrikines, resulting from the decellularization process as potential epitopes for a humorally mediated immune response.^5^

Although there is no current technique to silence the immune response after implantation of decellularized aortic homografts, currently implanted in the full-root technique, there might be the potential to reduce the amount of antigens by using a trimmed aortic valve and subcoronary implantation. This also holds the benefit of avoiding coronary reimplantation, which is necessary in the full-root technique and is—especially in smaller children—a known risk factor for coronary complications.^6^ To our knowledge, only 1 report is currently available on the clinical application of the subcoronary implantation for decellularized aortic homografts. In this case, significant early postoperative regurgitation was observed leading to early reoperation after 6 weeks and later to explantation of the decellularized aortic valve.^6^

Therefore, the aim of the current study was review technical aspects for subcoronary implantation of a decellularized aortic homograft valve in an ex vivo situation and in acute large-animal experiments. In the future, the same animal models will be used for long-term evaluation of this approach.

## Material and Methods

Ovine aortic roots were harvested from the slaughterhouse and decellularized using a published and authority-approved detergent-based decellularization protocol.^7^ Decellularized ovine aortic allografts (DOAA) were stored at 4° C in isotonic solution. The diameter of the aortic valve of these grafts varied between 20 and 25 mm. Fresh ovine hearts were harvested before the implantation from the slaughterhouse and prepared for implantation in dedicated surgical training devices.

Two cardiac surgeons (A.H. and A.W.) with a combined experience of >40 years in cardiac surgery, performed subcoronary DOAA implantation in 4 explanted ovine hearts, and results were compared with respect to procedural success and estimation of tissue quality.

Large-animal experiments were approved by the respective competent national authority (Niedersächsisches Landesamt für Verbraucherschutz und Lebensmittelsicherheit, reference No. 33.19-42502-04-24-00600). Experiments were performed within a dedicated large-animal facility (Medimplant GmbH) on the campus of the University of Veterinary Medicine Hannover, according current animal welfare legislation.

## Results

### Surgical Technique Ex-Vivo

**Figure 1:**
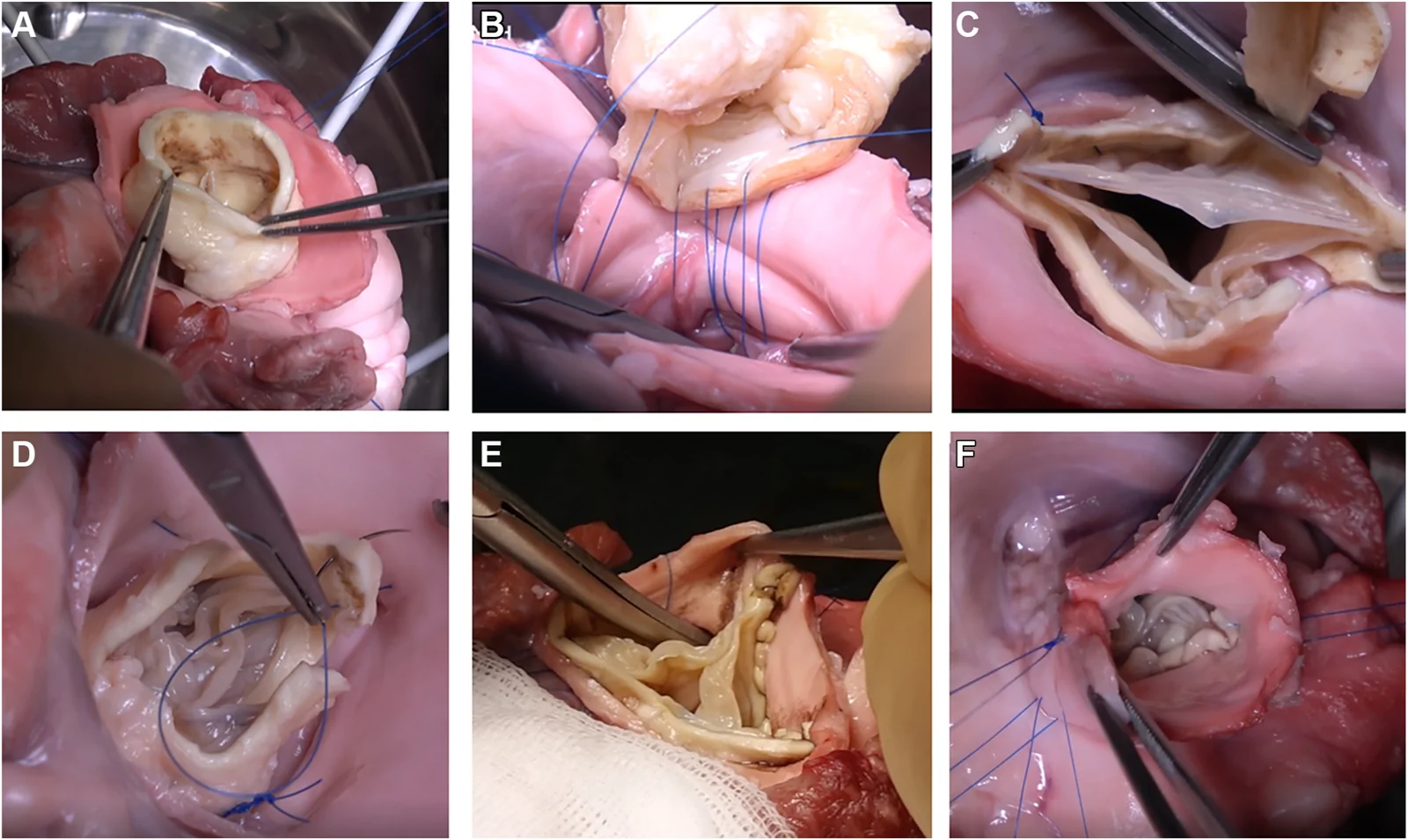
(A) Transverse complete aortotomy was followed by sagittal aortotomy down to the noncoronary sinus. The partially trimmed decellularized ovine aortic allograft (DOAA) was brought to the situs. (B) Running 5-0 Prolene sutures were used for proximal anastomoses and knotted with the aid of a threaded hook. (C) The DOAA aortic wall was further trimmed before the 3 valve commissures were sutured to the aortic wall of the recipient heart. (D) The 3 valve commissures were sutured to the aortic wall. (E) Running 5-0 Prolene sutures were used to attach the aortic valve between the respective commissures to the aortic wall. (F) Finally, the sagittal aortotomy was closed with a running suture.

Transverse complete aortotomy was followed by sagittal aortotomy down to the noncoronary sinus. Native cusps were excised, and the aortic annulus was sized with Hegar dilatators (20 and 22 mm). The decellularized allograft was trimmed. Although surgeon A did not excise the aortic root wall at this time, surgeon B exposed the commissures and aortic valve sinuses by removing the aortic wall of the DOAA. Running 5-0 Prolene (Ethicon) sutures were used for proximal anastomoses and knotted with the aid of a threaded hook. Surgeon A removed the aortic wall of the DOAA at this point before suturing the 3 valve commissures to the aortic wall of the recipient heart. Three running 5-0 Prolene sutures were used to attach the aortic valve between the respective commissures to the aortic wall. Finally, the sagittal aortotomy was closed with a running suture (Figure 1).

The 2 surgeons were able to perform the ex vivo subcoronary implantation of the DOAA without technical problems within 1 hour. Mechanical strength of the processed bovine DOAA appeared comparable to that of native aortic tissue.

**Figure 2:**
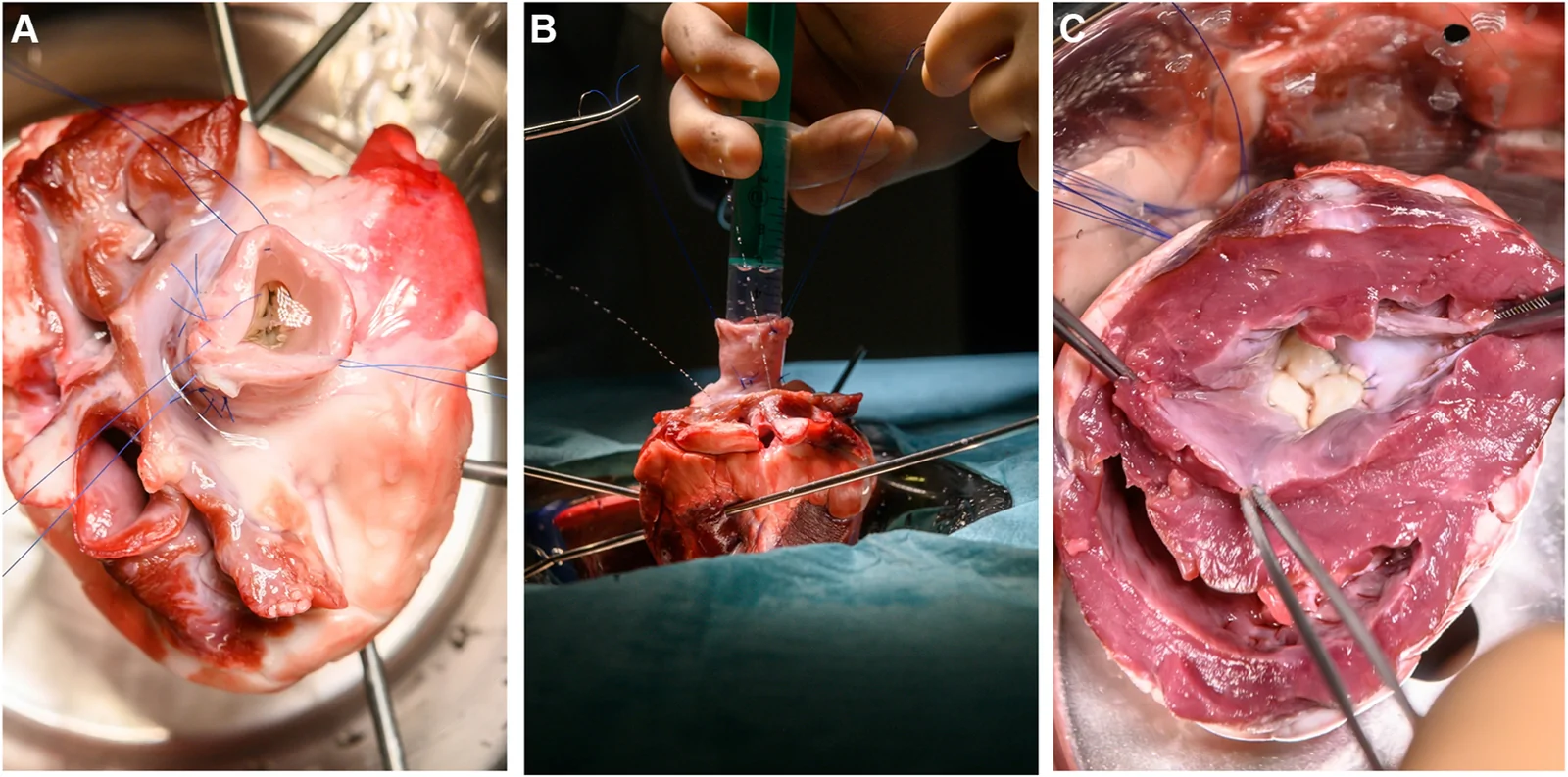
(A) Water test demonstrates full valvular competence. (B) Pressure water testing leads to fountain-like perfusion of the coronary arteries. (C) Subvalvular aspect of the subcoronary implanted decellularized ovine aortic allograft.

When asked to compare the tissue properties of DOAA to a human pulmonary valve used as an aortic autograft, both surgeons stated that there were no differences with respect to optics, haptics, trimmability, and sewability. Cusps of the subcoronary implanted grafts were highly mobile. and the opening diameter calibrated at Hegar 18 and 20 mm, respectively. Both implanted DOAAs were completely competent. Upon pressure, no leak into the left ventricle was noted, and the coronary arteries were perfused fountain-like (Figure 2; Supplemental Videos 1, 2).

In an additional ex vivo experiment, which was performed after the first in vivo animal experiment, 1 DOAA was implanted in subcoronary position using only 1 suture line, which, after trimming of the aortic wall sinuses, combined the annular suture line and the commissure/sinus suture line. To this end, the 3 commissures were sutured first into the aortic root at the height of the excised aortic valve. A running 5-0 Prolene suture attached the sinus level with the annular level and then to the aortic wall. This was performed for all 3 cusps and knotted to the commissure sutures with the aid of a threaded hook. Procedure time was ∼50% of the previously described 2-suture line technique with a perfectly integrated DOAA. There was also valvular competence with the modified unilayered suture technique.

### Surgical Technique in Vivo

Four adult female black-headed sheep were authorized for the establishment of the surgical technique in vivo. A left thoracotomy was used in all 4 animals. Exposition of the aortic valve in sheep is more difficult compared with humans due to the shorter ascending aorta.

In the first experiment, a DOAA was trimmed, as previously described, and implanted using the 2-suture line technique. After a long bypass time, the aortic valve showed severe regurgitation, which was even more pronounced due to a malpositioned aortic cannula, which had accidentally dislocated retrogradely into the ascending aorta. Learning from this experience, we switched to a single-suture line technique to achieve a faster and easier implantation.

**Figure 3:**
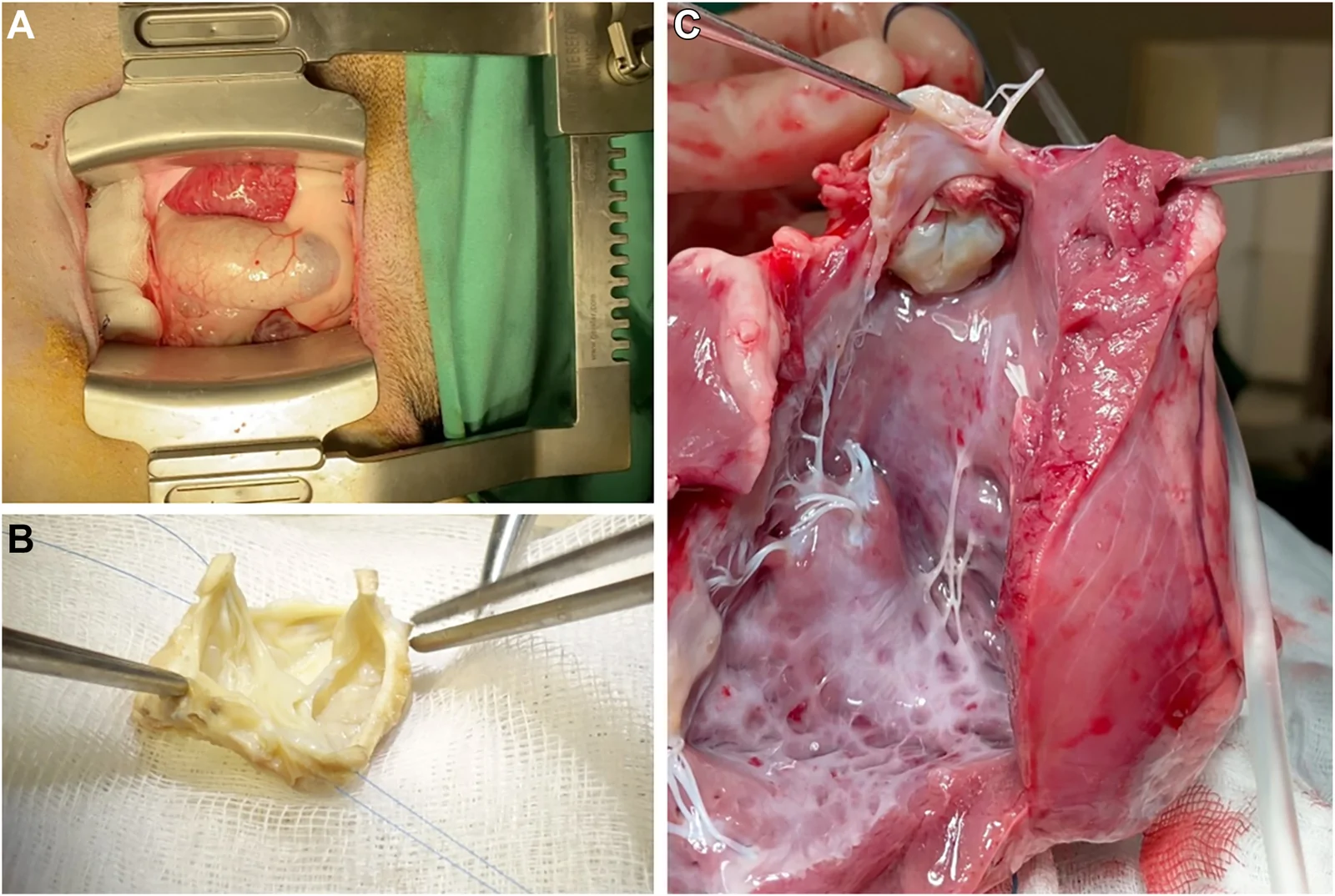
(A) Situs after left thoracotomy in adult sheep. (B) Extensively trimmed decellularized ovine aortic allograft before subcoronary implantation in single-suture line technique. (C) Prolapse of the aortic cusps leading to valvular incompetence.

The DOAA was extensively trimmed (Figure 3b). The commissures were sutured into the aortic root. Then running sutures of 5-0 Prolene attached the sinus level with the annular level and then to the aortic wall. This was performed for all 3 cusps and knotted to the commissure sutures with the aid of a threaded hook. Procedure time in the second sheep was ∼50% of the first procedure. At bypass end, the aortic valve showed prolapsing cusps, and consecutively severe aortic regurgitation was observed (Figure 3).

**Figure 4:**
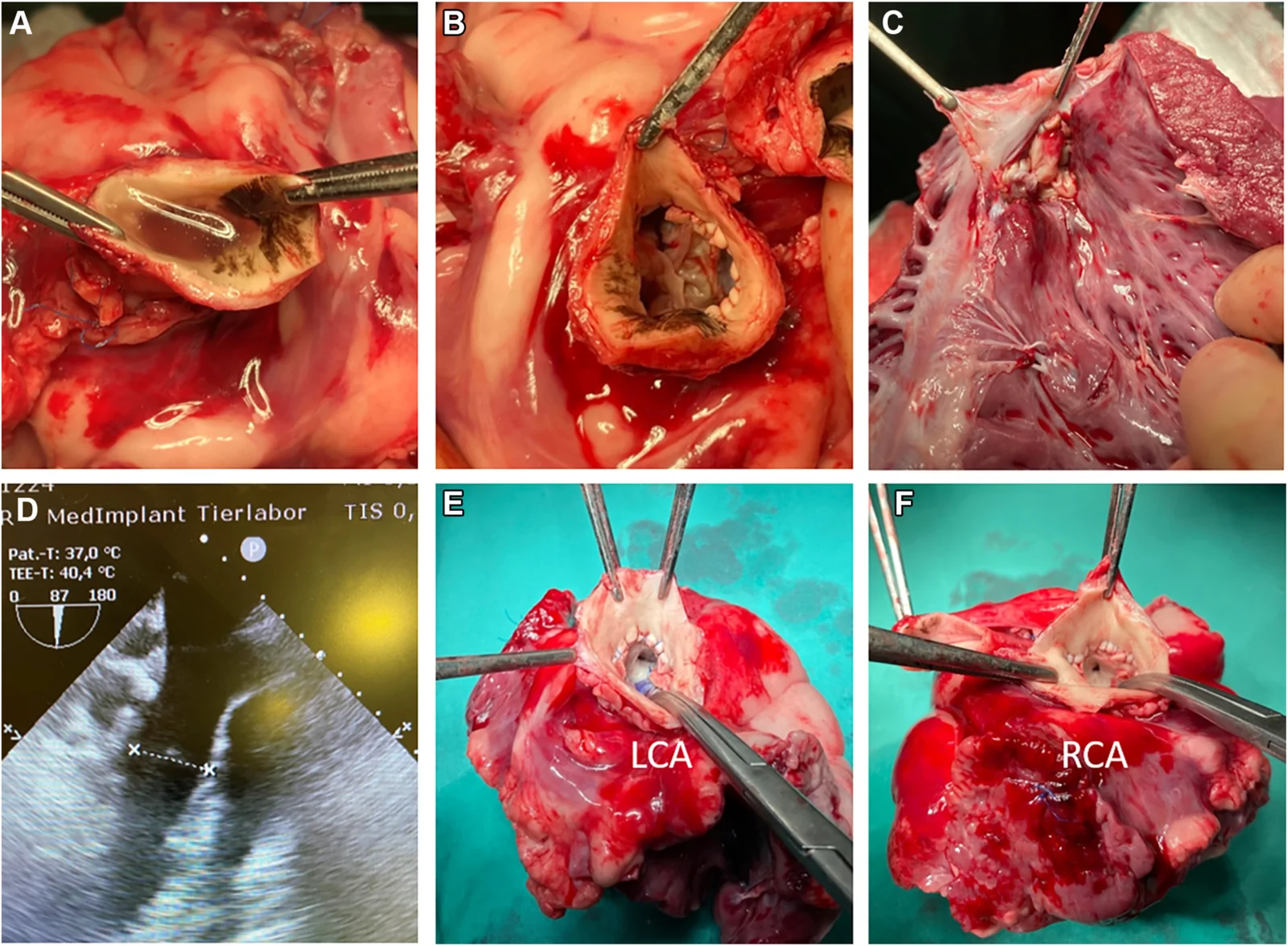
(A) Full valvular competence after subcoronary decellularized ovine aortic allograft (DOAA) implantation in sheep 4. (B) Supravalvular aspect after subcoronary DOAA implantation. (C) Subvalvular aspect after subcoronary DOAA implantation. (D) Unobstructed left ventricular outflow tract with a diameter of 20 mm. (E) Unobstructed left main coronary artery (LCA). (F) Unobstructed right coronary artery (RCA).

We identified the height of the reimplanted commissures as the leading problem. In the third and fourth animals, we positioned commissures at the highest feasible level and ensured that the cusp suture lines curved toward the commissures. With this technique, complete competence of the DOAA was achieved in these 2 animals, and transesophageal echocardiographic evaluation demonstrated excellent aortic valve function (Figure 4). Operative time was further reduced by parallel trimming of the DOAA and situs preparation by 2 lead surgeons.

## Comment

Options for aortic valve replacement in the pediatric age-group are limited. Mechanical valve replacement remains the main option for patients not requiring an aortic root procedure. For many surgeons, the pediatric Ross procedure is not appropriate due to the disadvantages of a 2-valve approach. The Ozaki procedure has recently gained interest in children with normal left ventricular outflow tracts, showing promising early results.^8^ However, the number of children who have been operated on remains small, and midterm results are still awaited.

We hypothesize that decellularized aortic homograft valves may add a further option for these pediatric patients when implanted in a nonroot procedure. Subcoronary implantation of decellularized homografts in normally sized aortic roots could eliminate the need for permanent anticoagulation compared with mechanical aortic valves and eliminate the need for coronary artery transfer. Coronary complications in children are, unfortunately, observed in ∼5% of pediatric root procedures and contribute significantly to morbidity and mortality.^4^

Here, we demonstrate the technical feasibility for subcoronary implantation of decellularized aortic allografts in a modified single-suture line technique. Ex vivo and acute animal tests in sheep showed symmetric implantations without aortic regurgitation and laminar flow across the subcoronary-implanted valves. Successful use of the single-suture technique requires accurate and extensive trimming of the decellularized allografts. Long-term animal tests are needed to confirm whether such an approach leads to long-lasting satisfactory valve function. These studies may also provide insight into the degree of recellularization and potential immunologic reactions toward the implanted allogenic components.

We believe that low-intensity immunologic reactions are the main reason for the inadequate regeneration of decellularized homografts, but the exact mechanisms of this immunologic response need to be clarified in future studies.

In conclusion, we have developed a technique for subcoronary implantation of decellularized aortic homografts, including acute large-animal experiments. Long-term animal experiments will hopefully provide further insight into durability and recellularization.
